# Molecular mechanisms of acquired resistance to tyrosine kinase targeted therapy

**DOI:** 10.1186/1476-4598-9-75

**Published:** 2010-04-12

**Authors:** J Rafael Sierra, Virna Cepero, Silvia Giordano

**Affiliations:** 1 Institute for Cancer Research and Treatment, University of Torino Medical School, 10060 Candiolo (Torino), Italy; 2 Present address: University Health Network, Ontario Cancer Institute and Princess Margaret Hospital, Toronto, ON, M5G 2M9, Canada

## Abstract

In recent years, tyrosine kinases (TKs) have been recognized as central players and regulators of cancer cell proliferation, apoptosis, and angiogenesis, and are therefore considered suitable potential targets for anti-cancer therapies. Several strategies for targeting TKs have been developed, the most successful being monoclonal antibodies and small molecule tyrosine kinase inhibitors. However, increasing evidence of acquired resistance to these drugs has been documented, and extensive preclinical studies are ongoing to try to understand the molecular mechanisms by which cancer cells are able to bypass their inhibitory activity.

This review intends to present the most recently identified molecular mechanisms that mediate acquired resistance to tyrosine kinase inhibitors, identified through the use of *in vitro *models or the analysis of patient samples. The knowledge obtained from these studies will help to design better therapies that prevent and overcome resistance to treatment in cancer patients.

## Introduction

The most common type of pharmacological anticancer treatment has been, for decades, conventional chemotherapy. This type of treatment does not discriminate between rapidly dividing normal cells and tumor cells, thus leading to severe systemic side effects, while attempting to reduce the tumor mass. In the last decade, the use of novel molecular targeted therapies has raised interest of both patients and clinicians. These treatments inhibit specific molecules that have a role in tumor growth or progression, and that are frequently altered in tumors but not in normal cells; thus, being more specific toward tumor cells, they are accompanied by reduced systemic toxicity [1]. Nowadays, targeted therapies represent an integrative approach to cancer therapy that has already led to important clinical results [2,3].

## Tyrosine Kinases

Tyrosine kinases have been identified as signaling molecules and prototypic oncogenes, and shown to play an important role in the development of many diseases, including cancer [4]. There is strong evidence that during tumor progression, the hyperactivation of tyrosine kinases leads to the continuous activation of downstream signaling cascades that block cellular apoptosis, promote cellular proliferation, and increase the nutrient/waste interchange by enhancing angiogenesis.

Receptor Tyrosine Kinases (RTKs) are single pass transmembrane proteins that account for almost two thirds of the genes coding for tyrosine kinases. RTKs possess a common functional kinase domain that is able to translate extracellular signals into active intracellular cues. Under physiological conditions, these receptors are activated only upon ligand binding [5]. Activation of the kinase is achieved by ligand-binding to the extracellular domain, which induces homo/hetero-dimerization of the receptors [6]. Activated receptors phosphorylate tyrosine residues outside their catalytic domain via cross-phosphorylation. This phosphorylation stabilizes the receptor conformation in an active state and creates phosphotyrosine docking sites for proteins which transduce signals within the cell [7,8].

In cancer, this mechanism of ligand-dependent activation can be bypassed by (i) overexpression of the RTK, which increases the dynamics of receptor homo/heterodimerization in the absence of the ligand [9-11]; (ii) by activating mutations, which stabilize the receptor active conformation [12]; or (iii) by autocrine stimulation. These mechanisms lead to cell autonomous activation of RTKs that drive proliferative and anti-apoptotic signals, contributing to transformation [7].

Non-Receptor Tyrosine Kinases (NRTKs), the second class of TKs, account for the remaining third of the approximately 90 known TKs and are critical signal transducers. Some examples include the well-known and well-characterized NRTKs Src, JAK, c-Abl and FAK. Interestingly, NRTKs were the first tyrosine kinases discovered [13-16]. Their involvement in cancer can occur through various mechanisms such as overexpression, mutation, and translocation; and therefore, many compounds have been developed attempting to inhibit their activity [17].

Treatments with tyrosine kinase inhibitors (TKIs), in some cases, have given promising results. However, most tumors treated with TKIs became resistant to treatment in a short time [18]. In other words, just as bacteria develop resistance to antibiotics, neoplastic cells can acquire new traits that render them more aggressive and able to survive in the presence of molecular inhibitors.

Clinical experience has shown that only a percentage of patients respond to targeted therapies, even if their tumor expresses the altered target. This *primary resistance *to treatment is often due to constitutive activation of downstream signal transducers [19-21]. Recently, many reports have evidenced that patients carrying activating mutations in effectors downstream of the targeted molecule account for the majority of the non-responsive patients [22,23].

Given that many patients are starting to benefit from tyrosine kinase inhibitors, including monoclonal antibodies and small molecule inhibitors, clinicians and basic researchers are now trying to unveil and understand the mechanisms through which neoplastic cells loose their ability to respond to these drugs (also known as *secondary resistance *or *acquired resistance*). Luckily, it appears that the majority of the resistance models developed *in vitro *are predictive of what is observed *in vivo *and can thus help researchers in identifying and studying this crucial clinical problem.

This review will attempt to provide an updated compendium of cellular modifications that contribute to acquired resistance to TKIs, highlighting the importance of preclinical studies of these drugs.

## Targeting Tyrosine Kinases

Many research groups, including ours, have shown that the inhibition of RTKs in neoplastic cells - by administration of monoclonal antibodies, interfering RNAs, and/or small kinase inhibitors (TKIs) - impairs cell proliferation and survival, inducing arrest of cell growth and apoptosis [24-28]. Based on these findings, many pharmaceutical companies have invested in designing or identifying new methods of inhibiting tyrosine kinases.

### Small molecule tyrosine kinase inhibitors

Pharmaceutical companies have focused their research on the development of small TKIs, some of which have received the approval of governmental drug administration agencies. Additional file 1 lists some TKIs currently approved or undergoing clinical trials. TKIs are small molecules that inhibit the enzymatic activity of the target protein. Most of these molecules can be categorized into four groups: (i) ATP-competitive inhibitors, which bind predominantly to the ATP-binding site of the kinase when this site is in the active conformation; (ii) inhibitors that recognize and bind to the non-active conformation of the ATP-binding site of the kinase, thus making activation energetically unfavorable; (iii) allosteric inhibitors, that bind outside of the ATP-binding site, modifying the tridimensional structure of the receptor and disrupting the interaction between the ATP and the kinase pocket; and (iv) covalent inhibitors, that bind irreversibly by covalently bonding to the ATP-binding site of the target kinase (reviewed in [29]).

While monoclonal antibody (mAb) therapy is particularly suited for extracellular (membrane-bound or secreted) targets, small-molecule kinase inhibitors are effective against both membrane-bound and intracellular targets. While both therapies have advantages and disadvantages when compared to each other, the major differences between monoclonal antibodies and small TKIs are the modality of administration, the bioavailability and half-life, and the mechanisms of resistance to the therapeutic agents [30-32]. (See comparison table 1).

**Table 1:** Major differences between monoclonal antibodies and small molecule tyrosine kinase inhibitors.

	mAb	Small molecule TKI
**Administration**	Intravenous	Oral or parenteral
**Target availability**	Must be extracellular	Extra/intra-cellular
**Cost**	US$ 4,200/month (trastuzumab)	US $1,800/month (gefitinib)
**Size**	~150,000 daltons	~400 daltons
**Diffusion**	Near vessels, surrounding tumor area; inefficient delivery	Easy to diffuse, translocate though plasma membranes, may reach brain tissues
**Toxicity**	Low toxicity	Mid-high toxicity
**Half-Life**	Days-weeks	<72 h
**Mechanism of Action**	Disrupt ligand-receptor or receptor-receptor (homo/hetrodimerization) interactions, receptor downregulation, induction of apoptosis	Bind to target kinase(s), inhibit phosphorylation and downstream signaling pathways. Induce apoptosis.
**Approval success rate**	18-24%	5%
**Mechanisms of resistance**	Protein Modifications: Switch of surface receptors. Shedding of the extracellular portion of the receptor. Expression of truncated receptors. Modification of receptor structure. Activation of downstream signaling pathways.	Genetic modifications: Point Mutations (Activating mutations). Amplifications (Target gene). Deletions. Protein Modifications: Overexpression of the target protein. Activation of alternative pathways. Overexpression of Multidrug resistance genes.

### Monoclonal Antibodies

Immunotherapy is based on the production of humanized monoclonal antibodies (mAbs) that bind with high specificity to secreted proteins or to the extracellular domain of membrane-bound proteins. The use of mAbs relies on the principle that most of the targeted molecules are expressed at higher levels on neoplastic cells, when compared to normal cells, where they play an important role in sustaining cancer progression. So far, there are several mechanisms described by which they exert their therapeutic effects; among them are: binding to the ligand or to the receptor, thus preventing ligand-receptor interaction [33,34]; disrupting receptor internalization [35], promoting receptor internalization [36], shedding of the extracellular portion of the receptor [36,37], preventing receptor dimerization and activation [38], and induction of apoptosis [39,40]. However, it is believed that each mAb acts through more than one mechanism. In addition, evidence has shown that activation of the immune response against the targeted tumor cells, upon recognition of the bound antibody, can also account for their biological activity [41]. Table 2 lists monoclonal antibodies directed again tyrosine kinases currently used in preclinical and clinical studies.

**Table 2:** List of Monoclonal antibodies approved by FDA or undergoing clinical trials.

Name of mAb	Commercial Name	Approval Year	Target Kinase	Mechanism of resistance
Bevacizumab (Genentech/Roche)	Avastin	2004	VEGFR	4
Cetuximab (ImClone and Bristol-Myers Squibb)	Erbitux	2004	EGFR	1,2,3,4
Panitumumab (Amgen)	Vectibix	2006	EGFR	
Trastuzumab (Genentech)	Herceptin	1998	ERBB2	2,4,5
IMC-A12 Cixutumumab (ImClone)		Phase II	IGF1-R	
AVE1642 (Sanofi-Aventis)		Phase I	IGF1-R	
Pertuzumab (Genentech)	Omnitarg	Phase III	ERBB2	
MetMAb (Genentech)		Phase I/II	MET	
IMC-1121B Ramucirumab (ImClone)		Phase III	VEGFR-2	
IMC-18F1 (ImClone)		Phase I	VEGFR	
AMG-102 Rilotumumab (Amgen)		Phase II	MET	

Antibodies common name is followed by () that denotes producer. Mechanisms of resistance: 1) overexpression of alternative RTK, 2) expression of receptor variants, 3) overexpression of target protein, 4) developed new signaling pathways, 5) structure modification

Monoclonal antibodies have been widely used in the clinic and have shown promising results, but unfortunately many patients relapse due to development of mechanisms of resistance. Information obtained from cellular models and relapsed patients has provided insights on how cells adapt to the treatment, by reducing the expression or modifying the structure of the target protein or activating alternative survival pathways [42].

## Mechanisms of resistance to TKIs

### Genetic modifications

**Figure 1:**
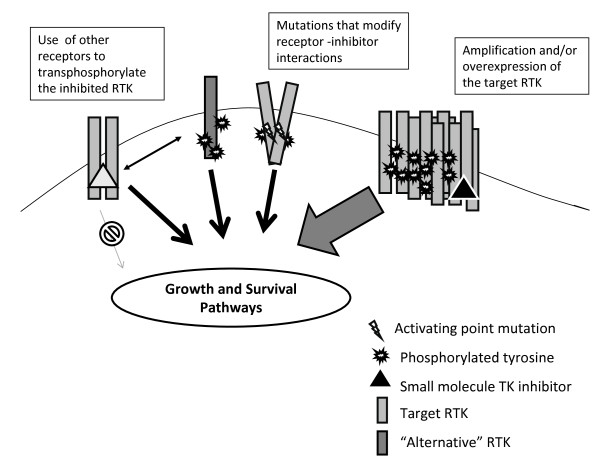
**Schematic summary of the main molecular mechanisms of acquired resistance to TKIs**.

Clinical and *in vitro *evidence have shown that cells treated with TKIs tend to acquire genetic modifications to overcome the inhibitory effects of these agents. Common mechanisms of resistance include, but are not limited to: point mutations, deletions and amplifications of genomic areas. A schematic summary of the main molecular mechanisms of acquired resistance to small molecules is represented in Figure 1.

#### Mutations

Mutations are common and occur frequently in rapidly dividing cancer cells. Point mutations are the most common mechanism of resistance to TKIs. The most frequent types of mutations are those that decrease the affinity of the drug for the target kinase domain, while maintaining its catalytic activity. Other mutations alter the amino acids surrounding the binding site of the drug and decrease the availability of the target region towards the inhibitor, without interfering with ATP binding [29]. Finally, some mutations increase the affinity of the kinase for ATP, decreasing the effectiveness of the ATP-competitive inhibitors [43].

The strongest evidence comes from imatinib, a small tyrosine kinase inhibitor that was found to bind with high affinity to c-Abl kinase. Imatinib is used to treat Chronic Myeloid Leukemia (CML) patients who express a constitutively active c-Abl tyrosine kinase, the BCR-ABL fusion protein. Imatinib abrogates the oncogenic function of BCR-ABL by binding the protein in its inactive state, thus preventing its autophosphorylation and, therefore, blocking the activation of downstream signal transducers. The use of imatinib has improved the life expectancy of CML patients, but major concerns have been raised for this and other TKIs by the rapid development of mechanisms of resistance. The majority of the CML patients in advanced stage (66%) and some in the chronic phase (5%) relapse after imatinib treatment, developing c-Abl dependent and independent mechanisms of resistance [44]. Approximately 30-50% of the relapsed patients acquire point mutations (around 90 distinct point mutations identified so far) that change the conformation of the c-Abl kinase, reducing or abrogating the ability of the compound to bind the c-Abl kinase domain [45-47]. This molecular mechanism of resistance has been supported also by structural studies which have shown that imatinib cannot efficiently interact with the ATP binding pocket in the mutated forms of BCR-ABL. When reports started to show that mutations in the kinase domain of c-Abl were present in relapsed patients, and experimental work showed that the mutant kinase was no longer inhibited by imatinib, second generation inhibitors, such as dasatinib [48], nilotinib [48,49], sunitinib [50], and bosutinib [51] were designed. These new molecules are able to recognize and bind BCR-ABL in different conformations, and are thus suitable for imatinib-relapsed patients. Dasatinib and nilotinib are able to interact with most of the mutated imatinib-resistant c-Abl forms, with the exception of the T315I mutant that changes the kinase and modifies several contact points between the drug and the kinase, while preserving the kinase activity [43,52,53]. The only inhibitor so far that has been proven to inhibit this mutant is the multikinase inhibitor KW-2449 [54]. However, CML patients who used these second generation inhibitors developed resistance by acquiring new mutations in the kinase domain [55].

Why do patients develop these mutations during treatment? There are reports that support the idea that the appearance of mutations in tumors after treatment with a specific TKI is the result of a process of selection of a pre-existing cell population. This theory implies that a small population of the tumor bulk *a priori *contains the mutation, which confers a primary resistance to these cells, therefore giving them a selective advantage. The bulk tumor mass is thus killed by the drug, allowing cells resistant to the TKI to grow. This theory is supported by the fact that some of these "resistance related mutations" can be found in a small percentage of tumor cells in patients that have not yet undergone targeted therapy [56-59]. On the other hand, other researchers believe that the high dependence of a cell on a specific oncogenic survival pathway forces genomic instability, allowing the induction of mutations that confer resistance to the inhibitor. This genomic instability can induce mutations either in the drug target or in other signal transducers that activate alternative pathways able to sustain cell viability. This theory has been supported by groups who have induced resistance to TKIs in imatinib-sensible CML cell lines cloned by limiting dilution; they have found the appearance of BCR-ABL gene amplification and of point mutations in the kinase domain that were not present in the original cells [60].

Further studies revealed that imatinib also binds with high affinity to the cKIT and PDGFR kinases, frequently activated in Gastrointestinal Stromal Tumors (GIST) [61]. GISTs are the first solid tumors in which a tyrosine kinase inhibitor was used as standard care. As these tumors often display mutations in the tyrosine kinase receptors cKIT and PDGFR, imatinib was used to inhibit their activity [62]. Like CML patients, 50-70% of GIST patients treated with imatinib develop secondary mutations within the cKIT gene, conferring a reduced drug binding affinity but still retaining the kinase activity [63,64]. To suppress the kinase activity of the resistant cKIT mutants, sunitinib was developed. As previously observed in patients treated with other inhibitors of second generation, imatinib-resistant GIST patients treated with sunitinib developed new mutations that made them again resistant to the new drug [65].

Gefitinib and erlotinib are small molecule TKIs targeting the Epidermal Growth Factor Receptor (EGFR) that have been used to treat tumors where this RTK is known to be altered. In particular, they have been used to treat non-small cell lung carcinomas (NSCLC) where EGFR is frequently overexpressed or activated due to point mutations [66]. According to a compendium of studies that include 1170 patients, more than 70% of NSCLCs with EGFR mutations respond to EGFR-TKIs, whereas only 10% of tumors without EGFR mutations do so. Unfortunately, upon treatment of these patients with gefitinib and erlotinib, two major mechanisms of resistance have been observed. The first is the appearance of a "resistance" point mutation in the kinase domain (T790M), observed in 50% of the gefitinib-resistant patients [67]. This mutation increases the affinity for ATP and weakens the affinity for ATP-competitive inhibitors [22,68]. On the other hand, the second mechanism is the activation of an alternative oncogene able to compensate for the inhibited signaling pathways [69,70].

Interestingly, *in vitro *models of acquired resistance to gefitinib, obtained by exposing gefitinib-sensitive cells to increasing concentrations of the drug, led to the appearance of the same mutations identified in patients. This has allowed scientists to study the mechanisms through which these mutations modulate sensitivity to the drug [71-75]. Lapatinib is another EGFR inhibitor, recently approved for treatment of breast cancer. This inhibitor has been designed to block receptor signaling by binding to the ATP-binding pocket of EGFR and ERBB2 kinase domains, thus preventing phosphorylation and subsequent downstream signaling from these two receptors [76]. Using a randomly mutagenized ERBB2 library *in vitro*, Trowe *et al*. were able to identify 12 mutations in the kinase domain of ERBB2 that could confer resistance to the inhibitor [77]. Moreover, this same work showed that a new generation inhibitor, EXEL-7647, is still active on all the mutants.

Similarly, activating mutations in the FLT3 RTK occur frequently in Acute Myelogenous Leukemia (AML). When AML patients were treated with PKC412, a staurosporin derivative able to inhibit FLT3's kinase activity, patients rapidly developed point mutations in the kinase domain of FLT3 that rendered the kinase less accessible to the inhibitor [78]. These same mutations had been previously foreseen by a computational predictive analysis and confirmed by *in vitro *data when Cools *et al*. identified possible mutations in residues conferring a high level of resistance to small molecules [79]. Recently, another cellular model has predicted new point mutations that confer resistance to FLT3 inhibitors such as SU5614, PKC412, and sorafenib. As the different FLT3 kinase inhibitors generated distinct, non-overlapping mutational profiles, the authors propose that a combination of FLT3 inhibitors might be useful to prevent the appearance of FLT3 resistance mutations [80].

As previously mentioned, another clinically approved TKI currently in use is sorafenib. This small molecular multikinase inhibitor, primarily targets BRAF and can inhibit several other TKs such as PDGFR, VEGFR 1-2, FLT3, and cKIT [81]. This multi-target drug possesses anti-tumoral and anti-angiogenic properties due to its broad blocking activity. The use of sorafenib, just as with other small molecule inhibitors, has caused so far a variety of mutations in PDGFR [82], FLT3 [80], and BRAF [83] that confer resistance to the treatment.

#### Gene Amplification

Gene amplification is a major mechanism of oncogenic activation [84]. Preclinical and clinical data have shown that the presence of either activating mutations in the kinase domain or gene amplification correlate with the best response to TKI [84,85]. Unfortunately, the selective pressure of the drug can drive further amplification of the target gene, thus leading to additional overexpression of the encoded protein. This idea originates from *in vitro *studies that have shown that highly amplified oncogenes are located in extrachromosomal acentromeric double minutes, and such cells undergoing "oncogenic stress" may undergo further gains due to advantageous unsymmetrical nuclear division [86]. These gains alter the stoichiometry of the drug-target interaction in favor of the target and result in its inefficient inhibition. This event has been observed in CML relapsed patients treated with imatinib, who displayed an increase in the *BCR-ABL *gene copy number [87]. In these patients, an increase in drug dosage is usually sufficient to restore responsiveness to the treatment. This same mechanism of resistance had been observed in an *in vitro *model where a CML cell line was treated for a long period of time with imatinib [42]. Likewise, the emergence of amplification of the target gene as a mechanism of resistance has been observed in two other cases where resistance cells amplified EGFR [88] or FTL3 [89] in response to inhibitors.

Another way through which gene amplification can mediate resistance to treatment is via amplification of genes that encode for critical transducers driving signaling pathways that can compensate for the signals lost due to target inhibition [69]. A notable example is the amplification of the *MET *gene, encoding for the receptor tyrosine kinase for hepatocyte growth factor, in a percentage of gefitinib-relapsed patients affected by NSCLC. These results perfectly correlated with those obtained in *in vitro *studies after treating sensitive NSCLC cell lines with progressively increasing doses of gefitinib or other EGFR inhibitors [57,58,70,90,91]. In these experiments, MET overexpression led to its constitutive activation by a ligand-independent mechanism, which later resulted in advantageous interactions with other EGFR family members, mainly ERBB3, and activation of downstream signals. Inhibition of MET, in this context, restored sensitivity to EGFR inhibitors [70].

#### Genomic Deletions

Other genomic alterations frequently observed upon TKI treatment are deletions. Khorashad and collaborators performed a genome-wide study comparing DNA samples from CML patients prior to imatinib treatment and after relapse. CGH analyses for all patients revealed that 28% of the copy number alterations were genomic deletions. Among the genes that were most frequently altered were those involved in the control of the MAPK signaling pathway [92].

Among the genes that are frequently deleted in human cancers are those encoding microRNAs (miRNAs). MiRNAs have emerged as a novel class of regulatory genes involved in human cancer [93,94]. Lacking the ability to encode a protein, these single-stranded miRNAs bind to imperfectly complementary sequences of encoding mRNAs, causing these mRNA sequences to be silenced or degraded, resulting in reduced levels of the protein encoded by the mRNA. Many reports have highlighted the relevance of these non-coding RNA's in human cancer, where they are frequently altered, more often as consequence of their deletion [95]. Various groups have reported cases where the deletion of miRNA regions has led to overexpression of the targeted RTKs, due to lack of down-regulation [95-97]. In this context, Seike and collaborators recently correlated high EGFR activation with high expression of mir-21 both in NSCLC patient samples and cell lines. They report that inhibition of EGFR by the small molecule AG1478 reduced the levels of this miRNA, concluding that the activation status of the receptor modulates the expression of this anti-apoptotic miRNA [98]. As it considered a growing field of interest, various groups have reported that miRNA expression can mediate resistance to different types of chemotherapy [99-102] (reviewed in [103]), and it is very likely that quite soon miRNAs will also be found to play a role in mediating resistance to TKIs.

### Modifications of protein expression

Cells seem to possess a broad repertoire of adaptive reactions that enable them to survive in many adverse conditions. One of the adaptive traits is the overexpression or the repression of genes that sustain cell viability [104]. Mahon *et al*. recently demonstrated that nilotinib-resistant CML cell lines were able to upregulate the expression of BCR-ABL, thus overcoming the inhibitory threshold of nilotinib [105]. Although this and other similar works lack evidence that the overexpression of the target protein is not due to gene amplification (also known mechanism of resistance to BCR-ABL TKI), this can be considered as a new mechanism of resistance.

This last response does not involve genetic alterations, but simply changes in gene expression, due to microenvironmental stress or to epigenetic modifications. It is known that the use of TKIs can lead to reduced blood flow, which in turn increases the incidence of hypoxic areas [106]. Moreover, hypoxia is known to upregulate HIF-1a, a protein that can promote the expression of many genes including the RTK MET, which is capable of sustaining the MAPK and PI3K survival pathways [107].

Likewise, epigenetic changes can also contribute to TKI resistance. For example, Noro *et al*. reported an *in vitro *model where lung cancer cells resistant to gefitinib displayed hypermethylation of the PTEN gene promoter; exogenous re-expression of this enzyme restores senstivity to the EGFR inhibitor [108].

### Activation of alternative pathways

Some cells can replace the lack of signal due to target inhibition by activating alternative pathways. The EGFR family of receptors has been shown to develop mechanisms of resistance by modifying the expression of several downstream effectors. For example, Pandya and collaborators developed a cellular model where colorectal carcinoma HCT116 cells, which depend on ERBB2 activity, lose their sensitivity to lapatinib. The major mechanism of resistance observed was the increased expression of MCL-1, and the decreased expression and activity of BAX and BAK [109], altogether leading to decreased apoptotic responses. Another proposed mechanism of resistance was reported by Xia *et al*. who showed that lapatinib-resistant breast cancer cells and lapatinib-treated patients displayed an increased level of the Estrogen Receptor and the transcription factor FoxoA3 [110]. Another example was recently reported by Turke *et al*. where EGFR-dependent cells stimulated with MET's ligand, HGF, were resistant both *in vivo *and *in vitro*, and such effect could be blocked by the use of MET inhibitors [57]. In a similar manner, McDermott *et al*. reported that MET-dependent NSCLC cells activate EGFR as a mechanism of resistance to PF2341066 (an irreversible MET kinase inhibitor) using an increasing dose resistant cellular model [111].

Another mechanism of resistance that was reported in NSCLC patients and in cell lines resistant to gefitinib treatment is the cross-talk between the EGFR/ERBB2 receptors and the IGF-1R receptor [112-114]. This mechanism of resistance relies on the fact that cells utilize IGF-1R to activate survival pathways that are able to promote growth [115]. One report shows that a prostate cancer cell line which became resistant to gefitinib displayed an increase of IGFII mRNA and IGF-1R protein phosphorylation [112,113]. Moreover, it was also published that a gefitinib-resistant lung squamous carcinoma cell line lost the production of IGFBP3-4 when compared to the parental cells; re-expression of these proteins restored the sensitivity to gefitinib's cytostatic effect [116].

The activation of an alternative kinase is known to overcome the inhibitory effects of small molecules. For example, GIST cells resistant to imatinib exhibited increased levels of the AXL receptor, that could in turn activate the AKT pathway and thus overcome c-KIT inhibition [97,117]. Two different groups have recently shown that in a cellular model of CML, TKI-resistant cells display activation of the Src kinase LYN; inhibition of this kinase by the use of dasatinib restores sensitivity to imatinib or nilotinib [105,118].

In a similar manner, the human myelomonoblastic cell line MV4-11, generated to be resistant to PKC412, displayed an up-regulation of anti-apoptotic genes and down-regulation of proapoptotic signals as well as genes that are involved in normal and malignant hematopoiesis [89].

Recently, Huang *et al*. reported that tumor xenografts resistant to sunitinib secreted higher amounts of IL-8 (proangiogenic factor known to be induced by several key regulators of cell survival and hypoxia) which at the same time positively correlated with a higher tumor vessel density [119,120].

Another commonly observed mechanism of resistance to TKI is the overexpression of survivin a member of the inhibitor of apoptosis family, encoded by the BIRC5 gene [110,121]. This cancer therapy candidate gene is overexpressed in a large variety of human tumors [122-125] and its expression is absent in terminally differentiated [126,127]. Survivin is known to inhibit caspase activation, and therefore, leading to negatively regulate apoptosis or programmed cell death, and it has been correlated with both accelerated relapse and chemotherapy resistance [128]. Xia *et al*. have demonstrated that overexpression of surviving can mediate resistance to lapatinib; such finding was observed by generating lapatinib-resistant breast cancer cells *in vitro *and correlating clinical observations [110].

### Mechanisms of Resistance related to drug influx/efflux

There are many mechanisms implicated in the decrease of the effective intracellular concentration of a drug, leading to lack of response to treatment. Among the most important resistance mechanisms are: increased drug influx/efflux and drug plasma sequestration. Other factors that can contribute to decreased drug delivery to tumors include irregular blood flow, defects in the structure and permeability of tumor vasculature and drug diffusion in the interstitium.

The occurrence of multidrug resistance (MDR) is a very frequent cause of failure of chemotherapeutic treatment in cancer patients. MDR proteins are transmembrane pumps responsible for the active efflux of a broad range of structurally unrelated molecules. This efflux can occur despite considerable concentration gradients at the expense of ATP depletion, resulting in decreased intracellular drug accumulation [129]. It is conceivable that TKIs may inhibit the function of ATP-binding cassette (ABC) transporters by recognizing their ATP-binding sites. In fact, some of these small molecules such as cediranib, lapatinib, and sunitinib have proven to be effective in reversing MDR associated to chemotherapeutics, by directly inhibiting the transport function of some ABC members. This ability renders them useful options for cancer combinational therapy [130,131]. The initial success of molecularly targeted therapies raised hope that newly developed agents would evade the general mechanisms of resistance that have reduced the efficacy of traditional anticancer drugs. However, ABC transporters related to MDR have emerged as key factors that regulate the intracellular concentrations of many small-molecule inhibitors. Drug transporters may be overexpressed in cancer cells, reducing intracellular drug concentrations, and may allow the evolution of point mutations that confer stronger drug resistance [132].

Mahone and collaborators demonstrated that imatinib-resistant cell lines overexpressed the P-glycoprotein (P-gp) efflux pump [133]. This concept was reinforced when imatinib sensitivity was restored when P-gp pumps were blocked by different inhibitors [134,135], or silenced using RNAi [136,137]. All this data indicates that P-gp is a likely candidate contributing to imatinib resistance, and some *in vitro *data suggests that this may also be true for resistance to nilotinib [105]. Dasatinib and sunitinib have been shown to be a substrate of both efflux proteins, ABCB1 and ABCG2 [138,139]. ABCG2 has also been shown to bind gefitinib with high affinity, causing an active extrusion of the inhibitor and thus preventing its biological activity [140].

In addition, multiple reports have provided evidence that deregulation of the organic cation transporter hOCT1 can impede the influx of imatinib. Using hOCT inhibitors on different imatinib-sensitive CML cells caused a reduced uptake of imatinib [141]. This finding was further supported by clinical data showing that patients who display a minimal response to imatinib also express a significantly lower amount of hOCT [142,143]. Therefore, intracellular drug levels depend in part on the differential expression of influx and efflux transporters, which are determinants of TKI resistance.

Another method by which tumors bypass the inhibitory effects of TKI is by the sequestration of such drugs by plasma proteins, such as the plasma protein-1 acid glycoprotein (AGP). It has been shown *in vitro *and *in vivo *that AGP binds to imatinib, and this binding decreases imatinib's ability to inhibit c-ABL in a dose-dependent manner [144], findings supported by clinical data [145,146].

## Mechanisms of resistance to monoclonal antibodies

Although monoclonal antibodies have given very good results in the clinic, the emergence of resistance is also frequently observed upon treatment with these agents. Several mechanisms of resistance have been observed in preclinical and clinical studies, mostly with antibodies that have already undergone FDA approval. In the case of monotherapy, preexistence of mutations in the MAPK or PI3K signaling pathways is one of the major causes of primary or intrinsic resistance. In 2009, the American Society of Clinical Oncology suggested that metastatic colorectal cancer (CRC) patients who displayed an alteration in codon 12 or 13 of KRAS should not be considered for monoclonal therapy [147]. This decision was based on multiple studies that have shown that activating mutations in KRAS [148-150], PIK3CA [19], BRAF [151] and loss of expression of PTEN [152-156] correlated negatively with cetuximab or panitumumab response (reviewed in [157]).

Patients undergoing monotherapy are also prone to develop secondary or acquired resistance to such treatment. So far, no mAb therapy has given rise to any point mutation in the target receptor or rearrangements in genomic regions. The mechanisms described up to now typically involve variations in protein expression. At least five modifications of this type have been shown to contribute to resistance to mAbs:

(i) Overexpression and aberrant phosphorylation of alternative RTKs attempting to overcome the inhibition of the targeted protein. In 2008, Wheeler *et al*. generated NSCLC and HNSCC cetuximab-resistant cell lines, such resistance was mediated by the increased expression of ERBB2, ERBB3, and MET which can interact with other EGFR family members contributing to their activation [35]. In a similar way, Lu *et al*. and Shattuck *et al*. have shown that cells can overcome trastuzumab inhibition by the activation of IGF-1R and MET, respectively [114,158-161].

(ii) The second known protein modification is expression of receptor variants. Sok and collaborators demonstrated that a mutant variant of EGFR (EGFRvIII), which lacks the ligand binding domain, is expressed in more than 42% of HNSCC. In their experiments, overexpression of EGFRvIII in HNSCC cells decreased in the inhibitory response to cetuximab [162].

(iii) The third protein modification involves the targeted protein; in this type of resistance, cells display an increased expression of the target receptor. Reports have shown that NSCLC cell lines resistant to cetuximab display an increase in EGFR protein levels due to a defective deregulation in the degradation pathways [35,163].

(iv) Activation of alternative pathways is another mechanism of resistance. It has been observed that cells resistant to either cetuximab or trastuzumab can develop a dependency on new signaling pathways either by triggering the same biological effects by interaction with other EGFR family members [35,164], or by association with other kinases such as Src [165]. Valabrega *et al*. reported that TGFα (an EGFR ligand) overexpression can contribute to resistance [166]. It is interesting to note that the overexpression of ligands is not a rare event, since patients and cell lines resistant to bevacizumab (a VEGF blocking antibody) cause tumor cells to secrete additional angiogenic factors (FGF [167], PGF [168], members of the notch ligand/receptor family [169]) to compensate for the lack of VEGF signaling [170,171].

Lastly, (v) the lack of interaction between the target and the mAb due to steric hindrance caused by the formation of complexes with other cell surface proteins, such as in the case of resistance to trastuzumab. It is known that the expression of MUC4, a membrane-associated mucin that contributes to the masking of membrane proteins, decreases the amount of trastuzumab that can bind to ERBB2 [172] When MUC4 was silenced in trastuzumab resistant cells, cells were once again sensitive to the mAb [173].

## Conclusions

New clinical and laboratory studies have suggested that multi-targeting approaches against neoplastic cells could help to increase patient survival and, possibly, reduce the emergence of cells resistant to single-target inhibitors [174]. This increased activity will have to be balanced by the expected increased toxicity due to the association of the drugs. Moreover, combination mAbs and multi-target small molecules could be also a very promising therapeutic approach [175,176].

Accumulating experimental and clinical evidences have supported the idea that targeted therapy should be reassessed. In particular, we should keep in mind that tumors are the result of multiple genetic lesions. Clinicians and researchers should not underestimate the capacity of tumors to easily adapt to new stress conditions, therefore inducing or selecting those cells that can better survive in the presence of an inhibitor.

## Abbreviations

CML: Chronic myelogenous leukemia; CRC: colorectal cancer; EGFR: epidermal growth factor receptor; GIST: gastrointestinal stromal tumors; HGF: hepatocyte growth factor; HNSCC: head and neck squamous cell carcinoma; mAb: monoclonal antibody; MET: hepatocyte growth factor receptor; NSCLC: non-small cell lung carcinoma; NRTK(s): non-receptor tyrosine kinase(s); RTK(s): receptor tyrosine kinase(s); TGFα: transforming growth factor alpha; TKI(s): tyrosine kinase inhibitor(s).

## Competing interests

The authors declare that they have no competing interests.

## Authors' contributions

JRS drafted and wrote the manuscript. VC helped writing the manuscript. SG corrected and finalized the manuscript. All authors read and approved the final manuscript.

## Supplementary Material

Additional file 1 **List of some small molecule TKIs approved by the FDA or currently undergoing clinical trials**. Click here for file
