# Supplementary material for: Molecular mechanisms of acquired resistance to tyrosine kinase targeted therapy
Source: Mol Cancer. 2010 Apr 12;9:75. doi: 10.1186/1476-4598-9-75 (PMC2864216; doi:10.1186/1476-4598-9-75)
Supplement: Additional file 1 — List of some small molecule TKIs approved by the FDA or currently undergoing clinical trials. [file 1476-4598-9-75-S1.DOC]

**Additional File 1.** List of some small molecule TKIs approved by the FDA or currently undergoing clinical trials.

| **Name of compound** | **Commercial Name** | **Approval/clinical trial phase** | **Target Kinase** | **Mechanism of resistance** |
| --- | --- | --- | --- | --- |
| ABT-869 [Linifanib]  (Genentech) |  | Phase II | VEGFR  PDGF |  |
| AEE788  (Novartis) |  | Phase I | EGFR, ERBB2, VEGFR, KDR, FLT-3 |  |
| AG 013736 [Axitinib]  (Pfizer) |  | Phase III | Pan- VEGFRs/ PDGFR |  |
| AMG 208  (Amgen) |  | Phase I | MET |  |
| AMG 706  (Amgen) |  | Phase III | Multitarget kinase inhibitor |  |
| AMN107 [Nilotinib]  (Novartis) | Tasigna | 2007 | BCR-ABL | 4 |
| ARQ197  (ArQule) |  | Phase II | MET |  |
| AV-951  (AVEO Pharmaceuticals) |  | Phase II | pan-VEGFRs |  |
| AZD0530 (AstraZeneca) |  | Phase II | Src, ABL |  |
| BAY 43-9006 [Sorafenib]  (Bayer) | Nexavar | 2007 | Multitarget kinase inhibitor | 1 |
| BMS-354825 [Dasatinib]  (Bristol-Myers Squibb) | Sprycel | 2006 | BCR-ABL, Src Family | 1,5 |
| CEP-11981  (Cephalon) |  | Phase I | Pan VEGFR/ Tie-2 |  |
| CEP-701 [Lestaurtinib]  (Cephalon) |  | Phase III | JAK2  FLT3 |  |
| CI-1033 / PD 183805 [Canertinib]  (Pfizer) |  | Phase II | pan EGFR family |  |
| CGP41251 [PKC412]  (Novartis) | Midostaurin | Phase III | FLT3 | 1,2 |
| CP-547632  (OSI Pharmaceuticals/ Pfizer) |  | Phase I/II | VEGFR2/ FGFRs |  |
| CP-690,550 (Pfizer) |  | Phase II | JAK3 |  |
| CYC116 (Cyclacel Pharmaceuticals) |  | Phase I | Aurora Kinases A/B; VEGFR2 |  |
| EKB-569 (Wyeth) |  | Phase I/II | EGFR |  |
| GSK1363089 (XL880)  (GlaxoSmithKline) |  | Phase II | MET / VEGFR2 | 1,2,3,4,5 |
| GSK572016 [Lapatinib]  (GlaxoSmithKline) | Tykerb | 2007 | EGFR/ ERBB2 | 1,5 |
| GW786034 [Pazopanib] (GlaxoSmithKline) | Votrient | Phase III | VEGFR |  |
| HKI-272 [neratinib ]  (Wyeth) |  | Phase I | pan EGFR family |  |
| JNJ-38877605  (Johnson & Johnson) |  | Phase I | MET |  |
| KW-2449  (Kyowa Hakko Kirin Pharma) |  | Phase I/II | Multikinase  FLT-3, BCR-ABL |  |
| MGCD265 (MethylGene) |  | Phase I | MET/ VEGFRs/RON |  |
| MK2461 (Merck) |  | Phase II | MET |  |
| MP470 (SuperGen) |  | Phase I | MET, RET, FLT-3, PDGFRα, multikinase inhibitor |  |
|  |  |  |  |  |
| OSI-774 [Erlotinib] (Genetech) | Tarceva | Phase III | EGFR |  |
| PF-02341066 (Pfizer) |  | Phase I | MET | 2,4,5 |
| PF-00299804 (Pfizer) |  | Phase II | Pan EGFR family | 4 |
| PF-04217903 (Pfizer) |  | Phase I | MET |  |
| PF-04554878 (Pfizer) |  | Phase I | FAK |  |
| PTK787/ZK222584  [Vatalanib] (Bayer Schering and Novartis) |  | Phase II | VEGFR2, PDGFR, cKIT |  |
| STI571 [Imatinib]  (Novartis) | Gleevec | 2001 | cABL, cKIT, PDGFR | 1,2,3,4,5 |
| SU11248 [Sunitinib]  (Pfizer) | Sutent | 2006 | Multitarget kinase inhibitor | 1,4,5 |
| TKI258 (Novartis) |  | Phase I/II | Multitarget kinase inhibitor |  |
| TSU-68, SU-6668 (Tiaho) |  | Phase I/II | VEGFR2, FGFR, PDGFR |  |
| XL184 (Exelixis) |  | Phase III | VEGFR2, MET and RET |  |
| XL228 (Exelixis) |  | Phase I | Src, BCR-ABL, IGF1R, FGFR |  |
| XL647 (Exelixis) |  | Phase I | EGFR, ERBB2, VEGFR2 |  |
| XL999 (Exelixis) |  | Phase I | VEGFR, PDGFR, FGFR, FLT-3, and Src |  |
| YN968D1 [Apatinib]  (Advenchen Laboratories) |  | Phase II/III | VEGFR |  |
| ZD1839 [Gefitinib] (AstraZeneca-Teva) | Iressa | 2005 | EGFR | 1,2,3,4,5 |
| ZD6474 [Vandetanib]  (AstraZeneca) | Zactima | Phase III | VEGFR/EGFR |  |

Compound common names are followed by [ ] stating compound’s common name, and by ( ) denoting the producer. Mechanism of resistance: 1) Point mutation, 2) gene amplification, 3) genomic deletions, 4) modification in protein expression, 5) activation of alternative signaling pathways.
